# Effect of CO_2_ Concentrations on Entomopathogen Fitness and Insect-Pathogen Interactions

**DOI:** 10.1007/s00248-024-02347-6

**Published:** 2024-01-23

**Authors:** Pascal Herren, Alison M. Dunn, Nicolai V. Meyling, Carlotta Savio, Helen Hesketh

**Affiliations:** 1 Faculty of Biological Sciences, University of Leeds, Leeds, LS2 9JT UK; 2 Department of Plant and Environmental Sciences, University of Copenhagen, Thorvaldsensvej 40, Frederiksberg, 1871 Denmark; 3 UK Centre for Ecology & Hydrology, Maclean Building, Benson Lane, Crowmarsh Gifford, Wallingford, Oxfordshire OX10 8BB UK; 4 Université Paris-Saclay, INRAE, Micalis Institute, Domaine de Vilvert, Jouy-en-Josas, AgroParisTech 78350 France; 5 Department of Plant Sciences, Wageningen University, Wageningen, 6708 PB The Netherlands

**Keywords:** Host-pathogen Interactions, *Tenebrio molitor*, *Bacillus thuringiensis*, *Metarhizium brunneum*, Insect Culture, Biocontrol

## Abstract

Numerous insect species and their associated microbial pathogens are exposed to elevated CO_2_ concentrations in both artificial and natural environments. However, the impacts of elevated CO_2_ on the fitness of these pathogens and the susceptibility of insects to pathogen infections are not well understood. The yellow mealworm, *Tenebrio molitor*, is commonly produced for food and feed purposes in mass-rearing systems, which increases risk of pathogen infections. Additionally, entomopathogens are used to control *T. molitor*, which is also a pest of stored grains. It is therefore important to understand how elevated CO_2_ may affect both the pathogen directly and impact on host-pathogen interactions. We demonstrate that elevated CO_2_ concentrations reduced the viability and persistence of the spores of the bacterial pathogen *Bacillus thuringiensis*. In contrast, conidia of the fungal pathogen *Metarhizium brunneum* germinated faster under elevated CO_2_. Pre-exposure of the two pathogens to elevated CO_2_ prior to host infection did not affect the survival probability of *T. molitor* larvae. However, larvae reared at elevated CO_2_ concentrations were less susceptible to both pathogens compared to larvae reared at ambient CO_2_ concentrations. Our findings indicate that whilst elevated CO_2_ concentrations may be beneficial in reducing host susceptibility in mass-rearing systems, they may potentially reduce the efficacy of the tested entomopathogens when used as biological control agents of *T. molitor* larvae. We conclude that CO_2_ concentrations should be carefully selected and monitored as an additional environmental factor in laboratory experiments investigating insect-pathogen interactions.

**Supplementary Information:**

The online version contains supplementary material available at 10.1007/s00248-024-02347-6.

## Introduction

CO_2_ (carbon dioxide) has the potential to affect host-pathogen interactions if either the host, pathogen, or both are affected by changes in CO_2_ concentrations. Numerous insect species are constantly exposed to CO_2_ concentrations above the atmospheric level, which is currently recorded as approximately 420 ppm (parts per million) [1]. Elevated CO_2_ concentrations can be a result of the respiration of insects [2, 3] or a product of increased microbial activity and subsequent accumulation in enclosed areas [4]. The CO_2_ concentration in soil air (inside soil pores), for example, is typically higher than the atmospheric CO_2_ concentration due to decreased gas exchange [4], hence soil-dwelling insect species are exposed to elevated CO_2_ concentrations in their environment. Furthermore, it is known that CO_2_ can accumulate in colonies of social insects reaching up to 60,000 ppm in leaf-cutting ant colonies [5], and 92,000 ppm in termite mounds [6]. Insects that are mass-reared for food and feed purposes can also be exposed to elevated CO_2_ concentrations because they are typically kept at high densities in closed systems [7], which facilitates the accumulation of CO_2_ [8].

The yellow mealworm, *Tenebrio molitor*, is an insect species that is increasingly being mass-reared to produce proteins and fats to feed livestock and for use in aquaculture [9, 10]. Respiration of *T. molitor* larvae produces approximately 60 g CO_2_ per kg of body mass per day or approximately 1,000 g CO_2_ per kg body mass gain [2]. Despite the utilisation of appropriate ventilations, CO_2_ is still likely to accumulate in production facilities of *T. molitor* [8, 11]; for example, in a closed experimental *T. molitor* rearing, CO_2_ concentrations reached up to 6,000 ppm [12]. The maximum permitted CO_2_ concentrations in production facilities are regulated by law in most countries to ensure the health and safety of employees [13]. For example, the long-term (8 h) exposure limit of CO_2_ concentration in the workplace is 5,000 ppm in many countries including the UK [14], the US [15], and countries belonging to the EU [16], which is more than tenfold higher than atmospheric concentrations.

Besides the use of *T. molitor* to produce feed, the yellow mealworm is also a global pest of stored grains and grain by-products [17]. The CO_2_ concentrations inside stored grains can exceed atmospheric CO_2_ concentration [18] and when there is microbial or insect activity, CO_2_ concentrations may increase even further [19, 20]. Various organisms (entomopathogens) such as bacteria, fungi, protists, nematodes, and viruses can infect *T. molitor* [21, 22]. Some of these entomopathogens are used as biological control agents against *T. molitor* in stored grains [23, 24] but at the same time, entomopathogens can also cause lethal or sublethal diseases in insects mass-reared for food and feed leading to economic losses in production systems [21]. Currently, there is a dearth of knowledge on how CO_2_ concentrations affect host-pathogen interactions in both mass-reared and wild insects [25]. Improving our understanding of the effects of CO_2_ on entomopathogens and their interactions with insect hosts will help to guide decisions of whether CO_2_ should be considered a relevant factor to include for insect-pathogen interaction experiments and in the design of insect mass rearing facilities.

CO_2_ is known to affect entomopathogenic organisms; for example, *Pseudoxylaria* spp., an entomopathogenic fungus infecting termites (*Odontotermes obesus*), showed reduced growth when exposed to elevated CO_2_ concentrations [6]. Furthermore, the number of conidia produced by different strains of the entomopathogenic fungal species *Metarhizium anisopliae*, *Isaria farinosa*, and *Beauveria bassiana* were generally decreased at 1,000 ppm CO_2_ compared to 350 ppm CO_2_ [26]. CO_2_ has also been found to affect the virulence of pathogenic organisms of humans [27]; in the human-pathogenic bacterium *Bacillus cereus*, for example, the expression of virulence genes was higher at elevated CO_2_ concentrations [28] and *Candida albicans*, a fungal pathogen of humans, switches from the monocellular to the more virulent filamentous growth at elevated CO_2_ concentrations [27]. Nevertheless, the impact of CO_2_ on the virulence of entomopathogenic organisms that can infect economically important insects remains unknown.

In this study, we examined the effects of CO_2_ on two entomopathogens, the bacterium *Bacillus thuringiensis*, and the fungus *Metarhizium brunneum*, which both naturally infect *T. molitor* [21, 22]. We used in vitro experiments and full-factorial bioassays to study interactions between CO_2_, insects, and pathogens. The pathogens were selected because both *B. thuringiensis* and *M. brunneum* can be found in stored grains [29–31]; grain products are both an important habitat of *T. molitor* and often used to feed *T. molitor* larvae in production systems [11]. Species of the genus *Metarhizium* are facultative entomopathogens, as these fungi can also colonize the rhizosphere of plants or live as saprotrophs [32, 33]. The impact of CO_2_ on fungal germination and growth in the external insect host environment is therefore highly relevant. On the other hand, *B. thuringiensis* is thought to only multiply inside the insect host while the environment (external to the insect host) constitutes a transition compartment for the spores and crystals without reproduction [34]. Therefore, the effects of CO_2_ on the viability and virulence of spores and crystals in the environment (e.g., soil or stored grains) are relevant to evaluate. The aims of this study were to assess the effects of elevated CO_2_ (4,500 ± 500 ppm) on: (1) the in vitro germination of conidia and mycelial growth of *M. brunneum*, (2) the in vitro viability and persistence of *B. thuringiensis* spores, and (3) the *in vivo* interactions between *M. brunneum* or *B. thuringiensis* and the larvae of *T. molitor*.

## Methods

All insect rearing and experiments took place in two separate 50-litre LEEC Culture Safe CO_2_ incubators adjacent to each other, one used for low [450 ppm (± 50 ppm)] CO_2_, and one used for high [4,500 ppm (± 500 ppm)] CO_2_ concentrations (see Supplementary methods). The low CO_2_ concentration corresponds approximately with ambient CO_2_ concentration, whereas the choice of the high CO_2_ concentration was based on maximum permitted concentrations for human safe working [13–16] and data from experimental setups [12]. To allow for maximum gas exchange in the Petri dishes in which the microorganisms were grown, the lids of all Petri dishes (unless otherwise stated) were elevated by adding 2 cm wide plastic strips between the lids and the lower dish. *Metarhizium brunneum* isolate KVL12-30 (culture collection of the Department of Plant and Environmental Sciences, University of Copenhagen, Denmark) and *Bacillus thuringiensis* serovar *morrisoni tenebrionis* 4AA1 *(Bacillus* genetic stock center, Ohio State University, USA) were used in experiments. The in vitro and the in vivo experiments were performed on three and two independent occasions, respectively.

### Germination and Growth of *M. brunneum*

The germination of *M. brunneum* conidia was assessed by adding 100 μl of 10^6^ conidia/ml (see Supplementary methods) on each of three replicate (per condition and time point) 10 ml SDAY/4 (16.25 g Sabouraud dextrose agar, 2.5 g yeast extract, and 11.25 g agar in 1 l dH_2_O) Petri dishes. The suspensions were spread using a Drigalski spatula and the Petri dishes were incubated at either low or high CO_2_ for 6, 8, 10, 12, 14, 18, or 24 h. Thereafter, 100 conidia were counted at three different locations on each Petri dish (300 conidia per Petri dish) and the numbers of germinated and un-germinated conidia were noted. A conidium was considered as germinated when it had a germ tube at least as long as the smallest diameter of the conidium.

The colony growth rates of *M. brunneum* at different CO_2_ concentrations were assessed by adding 2 μl of 10^6^ conidia/ml on the centre of each of ten replicate 30 ml SDAY/4 Petri dishes and subsequent incubation at either low or high CO_2_. The area of each colony was measured using a digital calliper on two perpendicular diameters, every second day for eight days, starting two days after the preparation of the Petri dishes. The average of the two diameters per colony was used as one data point for calculating the growth rate (mm/day) between days two and eight. Petri dishes that dried out before the end of the experiment were excluded from the analysis.

### Viability and Persistence of *B. thuringiensis*

The in vitro viability of *B. thuringiensis* spores was assessed by adding 100 μl of 10^3^ spores/ml (see Supplementary methods) to each of ten replicate 10 ml LB-Agar (lysogeny broth agar; 10 g tryptone, 5 g yeast extract, 10 g NaCl, and 15 g bacteriological agar in 1 l dH_2_O) Petri dishes. The suspensions were spread using a Drigalski spatula and the Petri dishes were incubated at either low or high CO_2_. At both CO_2_ concentrations, 100 μl of sterile dH_2_O was spread on each of three replicate 10 ml LB-Agar Petri dishes as controls (in the case of contamination this would be apparent on these Petri dishes). The numbers of colonies per Petri dish were counted after 24 h to calculate cfu/ml (colony forming units/ml; see Supplementary methods).

To measure in vitro persistence of *B. thuringiensis* spores, the method of Wood et al. [35] was adapted. Nine replicate autoclaved glass coverslips (22 × 22 mm) were placed inside an empty sterile Petri dish (three coverslips per Petri dish). On each coverslip, 100 μl of 6 × 10^5^ spores/ml (see Supplementary methods) were added and the Petri dishes containing the coverslips were incubated at either low or high CO_2_. Additionally, 100 μl of sterile dH_2_O was added on a separate coverslip in each Petri dish as a control (in the case of contamination this would be apparent on these coverslips). After two days the coverslips were transferred individually to 50 ml Falcon tubes containing 15 ml PBS (phosphate buffered saline) with Triton X-100 (0.1% v/v) and the tubes were put on an orbital shaker at 200 rpm at 25 °C for 15 min. Thereafter, 10 μl of the resulting suspensions were pipetted onto LB-Agar plates. By tilting the Petri dish on one side, the diluted suspensions ran down the media forming straight lines (three technical replicates on different Petri dishes were prepared). The average of the three technical replicates was used as one data point to calculate cfu/ml.

### In Vivo Bioassays

**Fig. 1:**
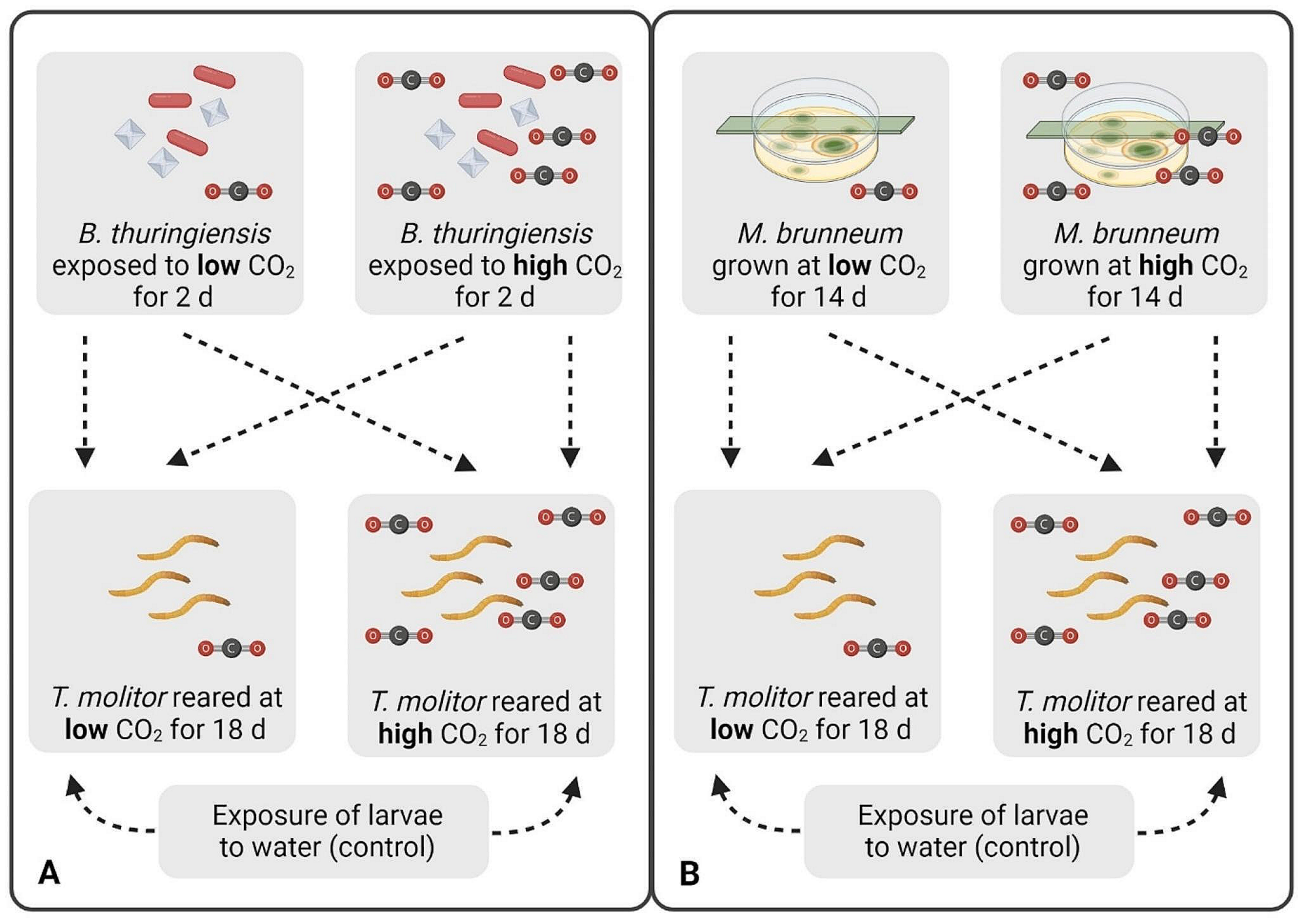
Schematic representation of the experimental design. **A** Larvae reared at either low or high CO_2_ for 18 days were exposed to *B. thuringiensis* previously exposed to either low or high CO_2_ for two days or to water as a control. **B** Larvae reared at either low or high CO_2_ for 18 days were exposed to *M. brunneum* grown at low or high CO_2_ for 14 days. The lids of the Petri dishes were elevated by adding a 2 cm wide plastic strip between the lower dish and the lid. **A, B** Each arrow represents one treatment (12 treatments in total, *n* = 5, 30 larvae per cup, two experimental repetitions). The survival, feed intake and weight of the larvae were assessed every second day for a period of 14 days after pathogen exposure. Figure created with BioRender (www.biorender.com)

*Tenebrio molitor* larvae were reared at either low or high CO_2_ concentrations for 18 days. *Bacillus thuringiensis* spores and crystals mixed in diet were exposed to either low or high CO_2_ concentrations for two days. *Metarhizium brunneum* was grown at either low or high CO_2_ concentrations for 14 days. The pathogens (100 μl of 4 × 10^9^ spores/ml per 100 mg diet for *B. thuringiensis* and 100 μl of 10^8^ conidia/ml per 100 mg diet for *M. brunneum*) were mixed into the larval diet [wheat bran (96% w/w) and dried egg white (4% w/w)] as described in the Supplementary methods. The larvae were exposed to lethal concentrations (previously determined in pre-experimental bioassays) of each pathogen separately in a full-factorial bioassay (*n* = 5, 30 larvae per cup). Furthermore, two groups of unexposed larvae (one at low and one at high CO_2_; *n* = 5, 30 larvae per cup) were prepared as control treatments (Fig. 1). The larvae per cup were weighed as a group the day before, and on the day of exposure to the pathogens. Two days after the start of the exposure to the pathogens, the larvae were transferred to fresh cups. The larvae and the remaining diet in each cup were separated from frass by using a sieve (0.5 mm) 2, 4, 6, 8, 10, 12 and 14 days after exposure. Larval mortality was also assessed on the same days after exposure and dead larvae were removed. The remaining diet and live larvae were weighed individually and new diet (0.6 × weight of live larvae) and water agar (1% w/v; 0.6 × weight of live larvae) were added on the same days as larval mortality was assessed (the value 0.6 was established in a pre-experimental bioassay to ensure that the larvae did not starve between feeding time points). The larvae from one cup treated with *B. thuringiensis* in the second experimental repetition were excluded from analysis because the cup was tipped over during the experiment.

### Statistical Analysis

Differences were considered as significant at *p* < 0.05 and data was only subjected to one-, two- or three-way ANOVAs (analysis of variances) when normality (QQ-plots) and homogeneity of variances (Levene test, *p* > 0.05) assumptions were satisfied. Tukey’s HSD (Honestly Significant Difference) tests were used to separate the means. All statistical analyses were performed using R v. 4.1.0 [36].

The effect of CO_2_ on *M. brunneum* conidia germination was described using a three-parameter log-logistic model ($y=\frac{d}{(1+{e}^{(b\left(\text{ln}\left(x\right)-\text{ln}\left(i\right)\right)})}$; where *y* = germinated conidia (%), *i* = inflection point (i.e., hours to 50% germination), *b* = slope, *d* = upper limit, *x* = time in hours) using the drc package [37]. The times to 50% germination at different CO_2_ concentrations were compared using the compParm function [37]. *Metarhizium brunneum* growth rates at different CO_2_ concentrations were analysed using a one-way ANOVA. Experimental repetitions were combined, as no interactive effect of repetition and CO_2_ was found in a previous two-way ANOVA. *Bacillus thuringiensis* spore persistence, spore viability, and density of larvae before the start of the in vivo assays at different CO_2_ concentrations were compared by implementing generalized linear mixed models with a negative binomial error distribution (used for over dispersed count data) using the lme4 package [38] with experimental repetition included as a random effect.

Mixed effects cox proportional hazards models were used to analyse the survival of the larvae in the in vivo assays (fixed effects: pathogen exposure, CO_2_ exposure of larvae, CO_2_ exposure of pathogens; random effects: experimental repetition, cup) using the coxme package [39]. Only significant fixed effects were retained in the final models and pairwise comparisons of treatments were performed using Tukey contrasts with single-step adjustment for multiple testing using the multcomp package [40]. The effect of CO_2_ on larval weight at the start of the experiment was analysed using a generalized linear mixed model with a gamma error distribution using the lme4 package [38] with experimental repetition included as a random effect. Weight gain per larva for the duration of the experiments and feed intake during exposure data were analysed separately for both experimental repetitions (Exp. 1 and 2) using two-way ANOVAs because interactive effects of experimental repetition and exposure of larvae or pathogens to CO_2_ were found in previous three-way ANOVAs.

## Results

**Fig. 2:**
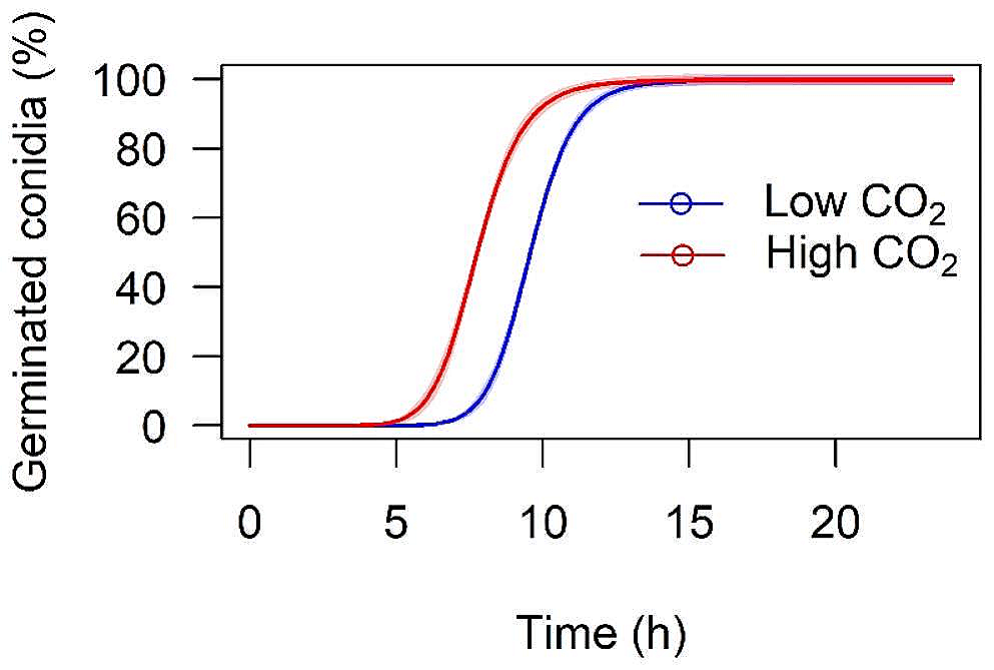
Three-parameter log-logistic models for germination of *M. brunneum* conidia over time (hours) at either low (*e* = 9.58 (confidence limits = 9.49 and 9.66); *b* = -12.74; *d* = 99.83) or high (*e* = 7.77 (confidence limits = 7.66 and 7.87); *b* = -9.83; *d* = 99.90) CO_2_ concentrations. The shaded areas represent the 95% confidence intervals

First, the effects of CO_2_ on different pathogen traits outside of the host were tested. The time to 50% germination of *M. brunneum* conidia was significantly lower at high CO_2_ compared to low CO_2_ (*t* = 26.07; *p* < 0.001). At both CO_2_ levels, the germination of conidia was > 99% after 24 h (Fig. 2).

*Metarhizium brunneum* colony growth rate was not affected by CO_2_ (Table 1). In contrast, *B. thuringiensis* spores incubated at high CO_2_ showed significantly lower viability than spores incubated at low CO_2_ (Table 1). Similarly, *B. thuringiensis* spore persistence was significantly decreased at high compared to low CO_2_ concentration (Table 1).

**Table 1:** *Metarhizium brunneum* colony growth rate, *Bacillus thuringiensis* spore viability and persistence at either low or high CO_2_ concentrations.^1^

	*M. brunneum* colony growth rate (mm/d ± SEM)^2^	*B. thuringiensis* spore viability (cfu/ml ± SEM)^3^	*B. thuringiensis* spore persistence (cfu/ml ± SEM)^4^
Low CO_2_	3.67 ± 0.04^a^	762.33 ± 61.11^a^	650.62 ± 98.07^a^
High CO_2_	3.73 ± 0.81^a^	682.00 ± 58.76^b^	213.58 ± 43.11^b^

^1^Means (± SEM) followed by different letters within a column indicate significant differences among the treatments. SEM, standard error of the mean; cfu, colony forming units

^2^*p* = 0.305, F = 1.077, d.f. = 1,43

^3^*p* < 0.001, χ2 = 16.971, d.f. = 1,58

^4^*p* < 0.001, χ2 = 13.419, d.f. = 1,52

**Fig. 3:**
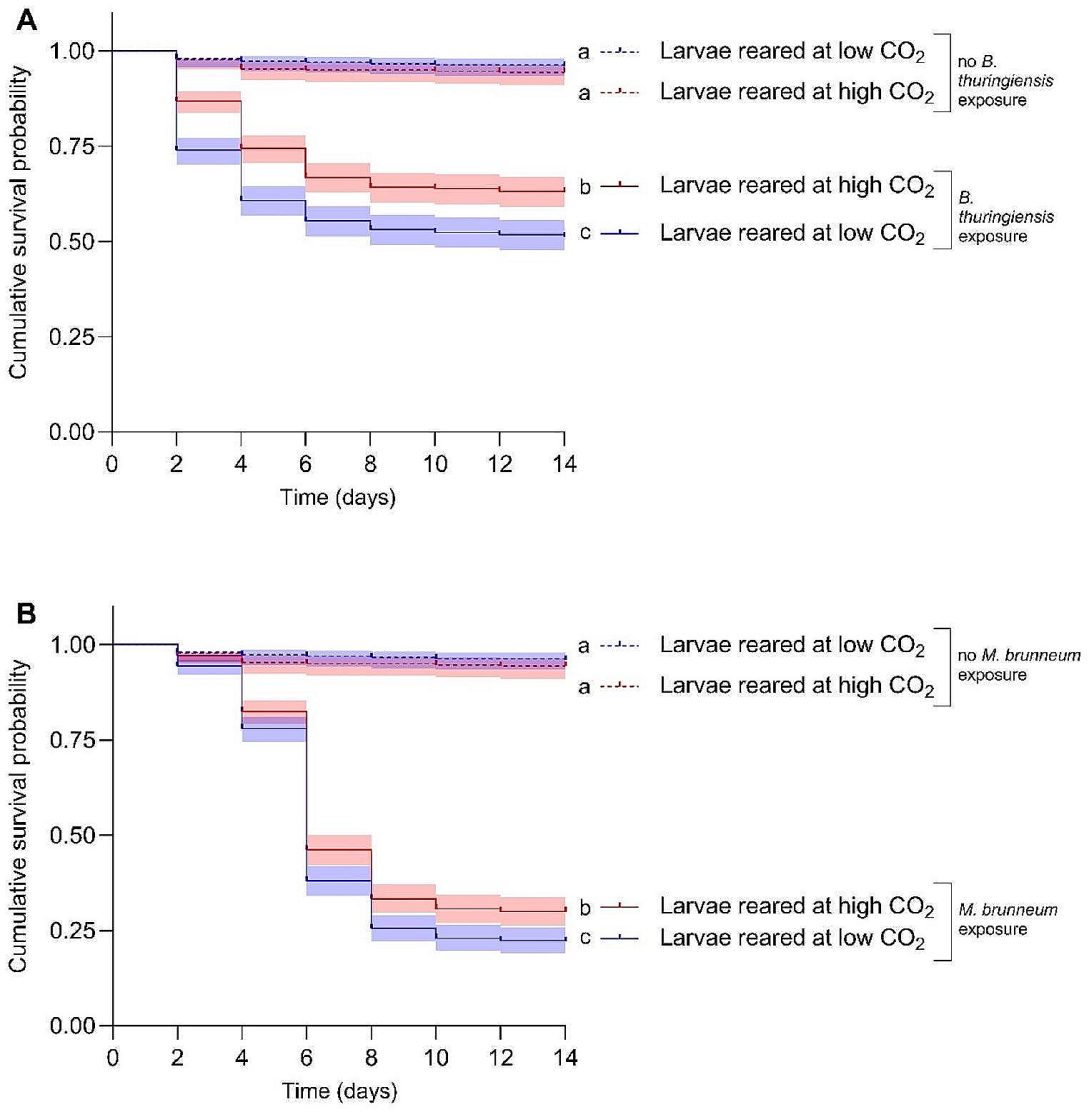
Survival of *T. molitor* larvae reared at either low (blue) or high (red) CO_2_ concentrations after exposure to pathogens for a period of 14 days. **A** Cumulative survival probability of larvae exposed to either low or high CO_2_ without (dotted survival curves) and with *B. thuringiensis* exposure (continuous survival curves). **B** Cumulative survival probabilities of larvae exposed to either low or high CO_2_ without (dotted survival curves) and with *M. brunneum* exposure (continuous survival curves). **A, B** Different letters to the right of the survival curves indicate statistical differences among treatments at *p* < 0.05. The shaded areas represent the 95% confidence intervals. Hazard ratios and *p*-values of fixed and random effects of the mixed effects cox proportional hazards models are displayed in Table 2. Figure created with GraphPad Prism version 9.3.1

To investigate host-pathogen interactions, full-factorial bioassays were performed in which the pathogens and the host were exposed to either low or high CO_2_ (Fig. 1). We tested the larval density in the two CO_2_ conditions before the start of the experiments to ensure that it did not affect our results. The larval density in the rearing containers at low and high CO_2_ was indeed not affected by CO_2_ (*p* = 0.311, χ2 = 1.026, d.f. = 1,6). Likewise, CO_2_ did not affect the weight of the larvae at the start of the experiment (*p* = 0.387, χ2 = 0.748, d.f. = 1,97). The germination rates of *M. brunneum* conidia were > 99% in all treatments and experimental repetitions. Larvae reared at high CO_2_ were significantly less susceptible (i.e., less likely to die) to *B. thuringiensis* (Fig. 3A; Table 2) and *M. brunneum* (Fig. 3B; Table 2) than larvae reared at low CO_2_ resulting in approximately 12 and 8% higher survival after 14 days, respectively. There was no effect of CO_2_ on survival of control larvae (*p* = 0.771, Fig. 3A, B). Moreover, exposure of the pathogens to different CO_2_ concentrations before exposure of the larvae did not affect the virulence of *B. thuringiensis* or *M. brunneum* (Table 2).

**Table 2:** Results of mixed effects cox proportional hazards models to analyse survival of *T. molitor* larvae^1,2^

***Bacillus thuringiensis*** **(*Bt*)**	**HR ± SE**	***p***
Exposure of larvae to *Bt*	11.505 ± 0.233	**< 0.001**
Exposure of larvae to CO_2_	0.693 ± 0.088	**< 0.001**
Exposure of *Bt* to CO_2_	0.992 ± 0.089	0.930
Cup	-	0.963
Experimental repetition	-	0.969
***Metarhizium brunneum*** **(*Mb*)**		
Exposure of larvae to *Mb*	31.352 ± 0.242	**< 0.001**
Exposure of larvae to CO_2_	0.816 ± 0.091	**0.025**
Exposure of *Mb* to CO_2_	0.954 ± 0.093	0.620
Cup	-	**0.011**
Experimental repetition	-	**< 0.001**

^1^HR ± SE (hazard ratio ± standard error) and *p-values* of fixed effects: Exposure of larvae to *Bt*, Exposure of larvae to CO_2_, Exposure of *Bt* to CO_2_, Exposure of larvae to *Mb*, Exposure of *Mb* to CO_2_; and *p*-values of random effects: Cup and Experimental repetition

^2^Bold *p*-values denote statistical significance at *p* < 0.05

**Fig. 4:**
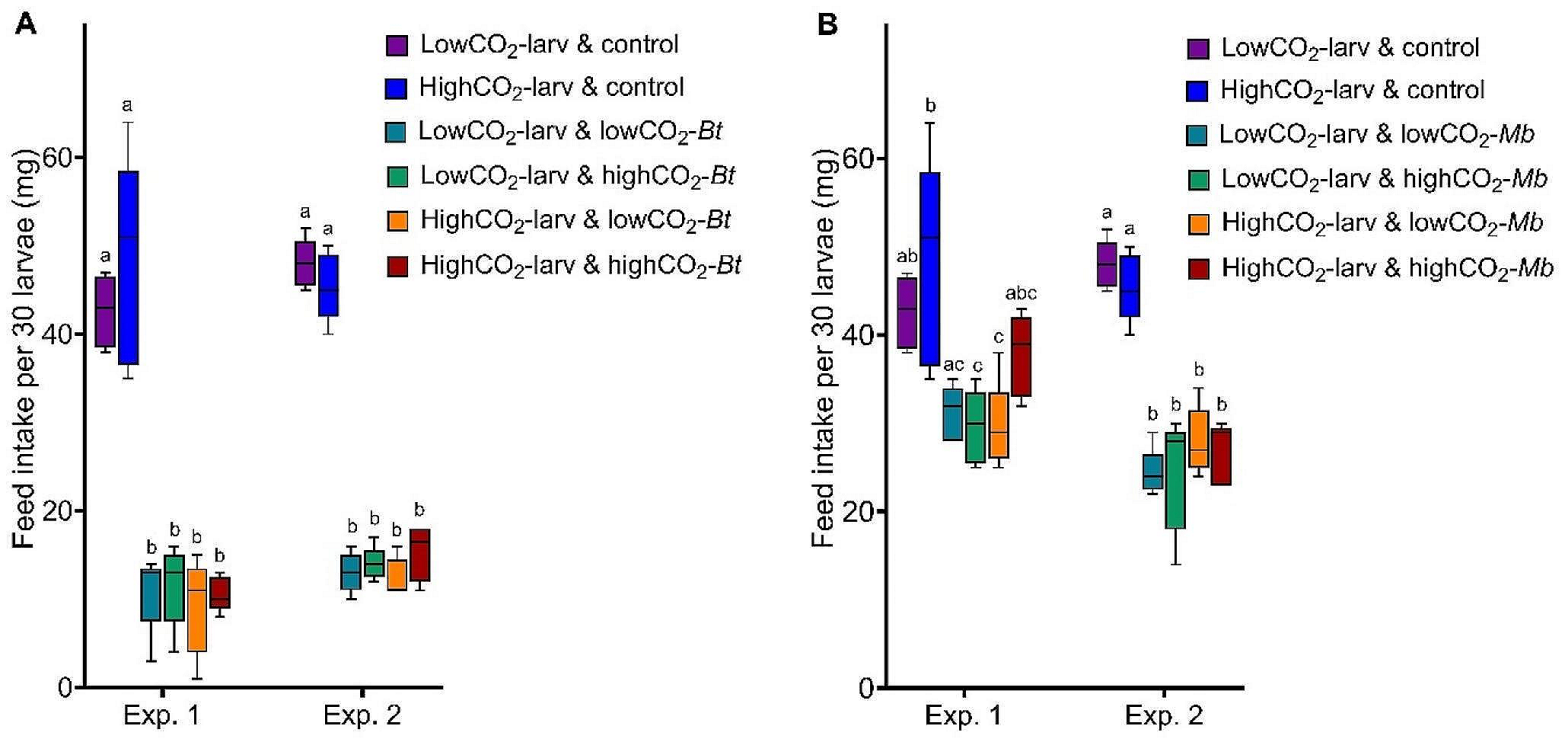
Feed intake per 30 larvae during exposure (two days) to the pathogens. **A** Feed intake during exposure to *B. thuringiensis* in Exp. (experiment) 1 and 2: control (no exposure to *B. thuringiensis*), lowCO_2_-larv (larvae exposed to low CO_2_), highCO_2_-larv (larvae exposed to high CO_2_), lowCO_2_-*Bt* (*B. thuringiensis* exposed to low CO_2_), highCO_2_-*Bt* (*B. thuringiensis* exposed to high CO_2_). **B** Feed intake during exposure to *M. brunneum* in Exp. (experiment) 1 and 2: control (no exposure to *M. brunneum*), lowCO_2_-larv (larvae exposed to low CO_2_), highCO_2_-larv (larvae exposed to high CO_2_), lowCO_2_-*Mb* (*M. brunneum* grown at low CO_2_), highCO_2_-*Mb* (*M. brunneum* grown at high CO_2_). **A, B** Boxplots show median, interquartile range, and minimum and maximum. Different letters above boxplots indicate statistical differences among treatments at *p* < 0.05 for each experiment separately. Degrees of freedom, F-values and *p*-values of the two-way ANOVAs are displayed in Table 3. Figure created with GraphPad Prism version 9.3.1

The effect of CO_2_ concentration and pathogen exposure on feed intake was measured during pathogen exposure. The feed intake per larva was reduced by *B. thuringiensis* exposure (Fig. 4A), but CO_2_ did not affect feed intake in either the control or *B. thuringiensis* exposed larvae (Fig. 4A; Table 3). Similarly, feed intake was reduced by *M. brunneum* exposure in the second iteration of the experiment, and in certain treatments of the first iteration (Fig. 4B). CO_2_ did not affect the feed intake during *M. brunneum* or control exposure (Fig. 4B; Table 3).

**Fig. 5:**
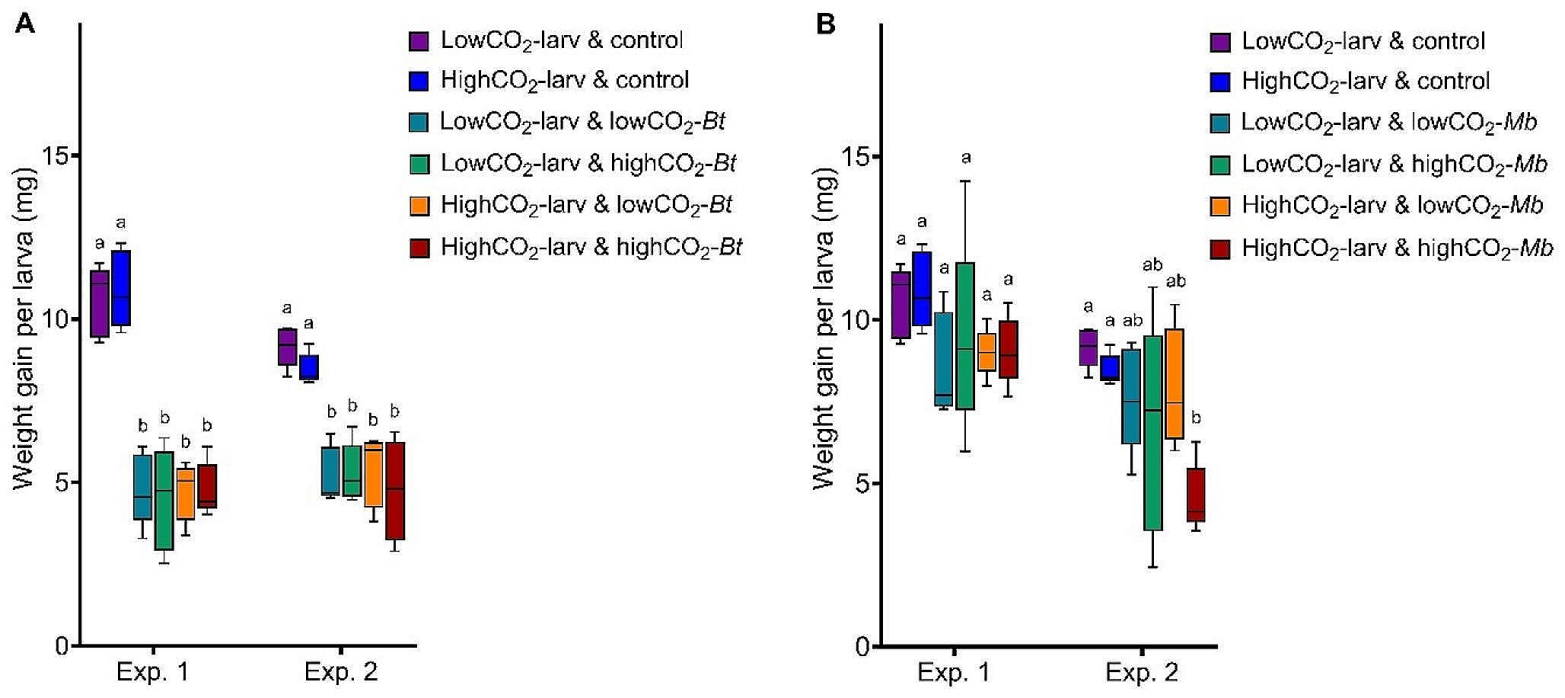
Weight gain per larva (mg) during 14 days after exposure to the pathogens. **A** Weight gain after exposure to *B. thuringiensis* in Exp. (experiment) 1 and 2: control (no exposure to *B. thuringiensis*), lowCO_2_-larv (larvae exposed to low CO_2_), highCO_2_-larv (larvae exposed to high CO_2_), lowCO_2_-*Bt* (*B. thuringiensis* exposed to low CO_2_), highCO_2_-*Bt* (*B. thuringiensis* exposed to high CO_2_). **B** Weight gain after exposure to *M. brunneum* in Exp. (experiment) 1 and 2: control (no exposure to *M. brunneum*), lowCO_2_-larv (larvae exposed to low CO_2_), highCO_2_-larv (larvae exposed to high CO_2_), lowCO_2_-*Mb* (*M. brunneum* grown at low CO_2_), highCO_2_-*Mb* (*M. brunneum* grown at high CO_2_). **A, B** Boxplots show median, interquartile range, and minimum and maximum. Different letters above boxplots indicate statistical differences among treatments at *p* < 0.05 for each experiment separately. Degrees of freedom, F-values and *p*-values of the two-way ANOVAs are displayed in Table 3. Figure created with GraphPad Prism version 9.3.1

Exposure of larvae to *B. thuringiensis* significantly reduced weight gain of the larvae over the course (14 days) of the experiments (Fig. 5A). However, weight gain was not affected by exposure of either the larvae or *B. thuringiensis* to different CO_2_ concentrations (Fig. 5A; Table 3). Exposure of larvae to *M. brunneum* did not affect the weight gain over the course of the experiment except for one treatment in the second iteration of the experiment (Fig. 5B). Furthermore, weight gain was not affected by exposure of either the larvae or *M. brunneum* to different CO_2_ concentrations (Fig. 5B; Table 3).

**Table 3:** Results of two-way ANOVAs to analyse feed intake of *T. molitor* larvae during the two days of pathogen exposure and individual weight gain of larvae during 14 days of individual experimental repetitions^1,2^

	Feed intake during exposure			Individual weight gain		
	d.f.	F	*p*	d.f.	F	*p*
***B. thuringiensis***, Experiment 1						
*Bt*	2,24	105.01	**< 0.001**	2,24	94.34	**< 0.001**
Larv-CO_2_	1,24	0.17	0.684	1,24	0.19	0.668
*Bt* * Larv-CO_2_	2,24	1.07	0.358	2,24	0.09	0.917
Experiment 2						
*Bt*	2,23	471.13	**< 0.001**	2,23	48.22	**< 0.001**
Larv-CO_2_	1,23	0.01	0.945	1,23	0.50	0.488
*Bt* * Larv-CO_2_	2,23	1.30	0.29	2,23	0.58	0.570
***M. brunneum***, Experiment 1						
*Mb*	2,24	16.24	**< 0.001**	2,24	3.96	**0.033**
Larv-CO_2_	1,24	3.24	0.084	1,24	0.04	0.837
*Mb* * Larv-CO_2_	2,24	1.69	0.207	2,24	0.18	0.838
Experiment 2						
*Mb*	2,24	87.37	**< 0.001**	2,23	8.54	**0.002**
Larv-CO_2_	1,24	0.58	0.452	1,23	2.03	0.167
*Mb* * Larv-CO_2_	2,24	1.64	0.216	2,23	1.10	0.350

^1^Abbreviations: *Bt*: *B. thuringiensis* treatment (either no *Bt*, *Bt* exposed to low CO_2_, or *Bt* exposed to high CO_2_); *Mb*: *M. brunneum* treatment (either no *Mb*, *Mb* exposed to low CO_2_, or *Mb* exposed to high CO_2_); Larv-CO_2_: exposure of larvae to either low or high CO_2_

^2^Bold *p*-values denote statistical significance at *p* < 0.05

## Discussion

In this study, elevated CO_2_ concentrations were found to decrease the viability and persistence of *B. thuringiensis* spores in vitro, whilst decreasing the duration of conidial germination of *M. brunneum*. Interestingly, exposure of the pathogens to different CO_2_ concentrations before infection did not affect the virulence of these entomopathogens toward *T. molitor* larvae, but larvae reared at elevated CO_2_ were less susceptible (i.e., less likely to die) to the pathogens than larvae reared at ambient CO_2_. These findings are important because *T. molitor* larvae are often exposed to CO_2_ concentrations above ambient conditions [8, 11, 18]. Here we show that CO_2_ levels affect the susceptibility of *T. molitor* to entomopathogens, which has implications for both mass-rearing of mealworms for food and feed purposes, and for biocontrol of this insect species. In addition to our main findings, we also found that CO_2_ did not affect the feed intake of the larvae during exposure to the pathogens and overall, did not affect the individual weight gain of the larvae. Investigating sub-lethal effects such as these is crucial, especially for the production of insects because a reduction in weight gain leads to economic losses, as the overall mass of insects produced is reduced.

It is challenging to put our study in context with other studies on CO_2_ because the few other studies that have been published investigating the effects of CO_2_ on insect-pathogen interactions either use lower (< 1,000 ppm) or significantly higher (> 50,000 ppm) CO_2_ concentrations than in this present study. To our knowledge, this is the first study to measure the effect of industrially relevant CO_2_ concentrations for the mass-rearing of *T. molitor* and other reared insect species. Elevated CO_2_ concentrations have been suggested to act as a cue promoting the germination of an entomophthoralean fungus (*Entomophaga maimaiga*) as CO_2_ concentrations might be elevated near the insect cuticle [41]. This increased germination of fungal conidia is in accordance with our study. However, decreased germination and mycelial growth of a hypocrealean fungus (*B. bassiana*) were reported as a result of a very high CO_2_ concentration (400,000 ppm) [42]. Similarly, 50,000 ppm CO_2_ decreased the mycelial growth and sporulation of *M. brunneum*, *Aspergillus* sp., and *B. bassiana in vitro* [43]. Moreover, it was proposed (without statistical analyses) that the growth rates of different *M. anisopliae* strains are either positively or negatively affected by elevated CO_2_ (650 and 1,000 ppm) [26]. We, in contrast, did not find an effect of CO_2_ at industrially relevant concentrations on the growth rate of *M. brunneum in vitro*.

This is, to our knowledge, the first study that measures the direct effects of elevated environmental CO_2_ on the persistence and viability of a bacterial entomopathogen. However, it is known from other species that CO_2_ can reduce bacterial growth [44]. We found that the persistence of *B. thuringiensis* spores was almost three times lower at elevated CO_2_. Surprisingly, there was no effect of exposure of *B. thuringiensis* to elevated CO_2_ on the subsequent virulence in the insect host. This could be because the crystals of *B. thuringiensis* that are essential for the infection process might not be affected by CO_2_. Moreover, we speculate that the spores kept at elevated CO_2_ could have been only temporarily inactivated (dormant) and might be reactivated in the host. It has been shown for other species of the *Bacillus* genus that suboptimal thermal and pH conditions during incubation can increase the time to germination of spores [45].

Interestingly, we could not detect any sublethal effects of elevated CO_2_ on the larvae. In contrast, in a study by Li et al. [12], *T. molitor* larvae reared in a closed system had a lower weight gain compared to larvae reared in an open system, which was argued to be due to higher CO_2_ concentrations in the closed system [12]. However, these differences could also have been due to other factors such as different relative humidity or different concentrations of other gases in the two systems. It is important to note that elevated CO_2_ concentrations may be more detrimental to insects when the relative humidity is low, because elevated CO_2_ forces the insects to keep their spiracles open, which can result in water loss [46].

Our study supports prior findings by Borisade & Magan [26] who exposed desert locusts (*Schistocerca gregaria*) and house crickets (*Acheta domesticus*) to elevated CO_2_ concentrations (1,000 ppm). The authors suggested that *S. gregaria* and *A. domesticus* kept at elevated CO_2_ showed increased survival and lethal times, respectively, when exposed to *B. bassiana*, although this was not statistically validated [26]. In contrast to these findings, the survival of red flour beetles (*Tribolium castaneum*) exposed to *B. bassiana* was significantly decreased at very high CO_2_ concentrations (440,000 ppm) [42]. Due to our experimental design, we can disentangle the effects of CO_2_ on the interactions between the pathogens and *T. molitor*, demonstrating that previous exposure of the pathogens to elevated CO_2_ did not affect the virulence of the pathogens, but that rearing the larvae at elevated CO_2_ decreases the susceptibility of the larvae to the pathogens. One possible explanation is that CO_2_ may affect the insect immune response. For example, in *Drosophila melanogaster* the production of antimicrobial peptides was inhibited by CO_2_ (130,000 ppm) correlating with increased susceptibility to bacterial infections [47]. Moreover, in *T. castaneum* CO_2_ increased the production of benzoquinones [48] (a quinone that is also produced by *T. molitor* [49]), which inhibit *B. bassiana* [50]. The mechanism underlying the decreased susceptibility of *T. molitor* to pathogens at elevated CO_2_ concentrations remains to be investigated. Moreover, it would be beneficial for the production of *T. molitor* and other mass-reared insect species to investigate the CO_2_ concentrations *T. molitor* is evolutionarily adapted to in order to optimise rearing conditions. *Tenebrio molitor* might be adapted to elevated CO_2_ concentrations whereas other species may be adapted to different CO_2_ concentrations.

Here, we demonstrate that CO_2_ directly affects a bacterial and a fungal entomopathogen in vitro and their in vivo interactions with an insect host. Based on these results, we conclude that the tested elevated CO_2_ concentration (4,500 ± 500 ppm) in *T. molitor* mass-rearing systems is beneficial for larvae exposed to the tested pathogens by increasing larval survival. Furthermore, we did not find any sublethal effects of CO_2_ on *T. molitor* larvae that would affect the overall productivity of the mass-rearing system. For biocontrol of *T. molitor*, our results indicate that the efficacies of the two tested entomopathogens may be lowered at elevated CO_2_ concentrations, which has implications for understanding the reliability of biocontrol of storage pests. To ensure meaningful conclusions, we suggest it is crucial to consider CO_2_ effects (i.e., through monitoring and using pertinent CO_2_ concentrations) when studying any insect pathogen systems that are likely to be exposed to elevated CO_2_ in their natural or artificially maintained environments.

### Electronic Supplementary Material

Below is the link to the electronic supplementary material.

Supplementary Material 1

## Data Availability

The datasets generated for the current study are available from the corresponding author on reasonable request.
