# Supplementary material for: Effect of CO2 Concentrations on Entomopathogen Fitness and Insect-Pathogen Interactions
Source: Microb Ecol. 2024 Jan 23;87(1):34. doi: 10.1007/s00248-024-02347-6 (PMC10805855; doi:10.1007/s00248-024-02347-6)
Supplement: Supplementary file 1 — Supplementary Material 1 [file 248_2024_2347_MOESM1_ESM.docx]

**Supplementary Material**

Supplementary Methods

*Control of environmental conditions*

To assure appropriate ventilation, the LEEC Culture Safe CO_2_ incubators were fitted with 40 mm ELUTENG USB fans through the access hole at the back of the incubators, which were turned on for 15 min every 45 min. The CO_2_ concentrations were monitored every 15 min using Rotronic CL11 loggers. The incubator used for low CO_2_ was set to 400 ppm whereas the incubator used for high CO_2_ was set to 5,000 ppm. The actual CO_2_ concentrations measured were 450 ppm (±50 ppm) and 4,500 ppm (±500 ppm) CO_2_ in the incubators set to low and high CO_2_, respectively. Moreover, the temperature and relative air humidity were monitored every 15 min using two EasyLog EL-SIE-2 loggers per incubator positioned on the top and bottom shelves. The temperature in both incubators was maintained at 28°C (±0.5°C) and the relative humidity at 75% (±10%) in complete darkness.

*Preparation of* Metarhizium brunneum *suspension*

*Metarhizium brunneum* was grown on Petri dishes (9 cm diameter, triple vented) containing 30 ml SDAY/4 media sealed with Parafilm^TM^ in the incubator at low CO_2_ for 14 days. Thereafter, conidia were harvested using a Drigalski spatula after adding 5 ml TritonX-100 (0.05% v/v) on each Petri dish. The conidia suspensions were dispensed into 50-ml Falcon tubes and centrifuged at 3,000 rpm (1,872 *g*, Eppendorf Centrifuge 5810 R) for 3 min. After disposing of the supernatant, additional TritonX-100 (0.05% v/v) was added, and the centrifuging was repeated using the same settings. The stock suspension was obtained by disposing of the supernatant and adding fresh TritonX-100 (0.05% v/v). The stock suspension was serially diluted to obtain a 1,000 times diluted suspension and the concentration of the conidia was enumerated in a 0.2 mm Fuchs-Rosenthal hemocytometer. The final stock concentration was diluted to achieve the concentrations used in the respective experiments.

*Assessment of conidial viability*

To assess conidial viability, 100 μl of a 10^6^ conidia/ml suspension was spread on each of three replicate Petri dishes (9 cm diameter, triple vented) containing 10 ml SDAY/4 media and sealed with Parafilm^TM^. After 18 hours incubation time at 28°C, 300 conidia were counted on each Petri dish using a light microscope at 100 times magnification. If the germ tube of a conidium was equal to, or longer than its smallest diameter, it was considered as germinated during the counting.

*Preparation of* Bacillus thuringiensis *suspension*

*Bacillus thuringiensis* was grown overnight on Petri dishes (9 cm diameter, triple vented) containing LB-Agar at 30°C in complete darkness. One colony was then picked from the plate using a sterile inoculation loop and added to a 500 ml Erlenmeyer flask containing 50 ml liquid HCT medium (5 g tryptone, 2 g bacto casamino acids, 6.8 g KH_2_PO_4_, 0.1 g MgSO_4_, 0.002 g MnSO_4_, 0.014 g ZnSO_4_, 0.15 g CaCl_2_ and 0.022 Ammonium ferric citrate in 1 l dH_2_O) sealed with Parafilm^TM^. The culture was incubated on a platform shaker at 200 rpm at 30°C for four days. Thereafter, the bacterial suspension was poured into a 50 ml Falcon tube and centrifuged for 10 min at 3,900 rpm (3,231 *g*, Eppendorf Centrifuge 5810 R) at 4°C. The supernatant was discarded and 20 ml sterile dH_2_O was added. This washing step was repeated twice thereafter with the settings mentioned above. The obtained stock suspension was incubated at 75°C for 10 min to obtain a spore and crystal suspension without viable vegetative cells. Spores and crystals were confirmed to be present through examination under a light microscope at 1,000 times magnification. Thereafter a ten-fold serial dilution was prepared. Subsequently, 10 μl of each of the six diluted suspensions (10^4^ - 10^9^) were pipetted in triplicate onto 10 ml LB-Agar plates (three dilutions per Petri dish). By tilting the Petri dish on one side, the diluted suspensions ran down the media forming straight lines. All Petri dishes were incubated at 28°C for 16 hours and the stock suspension was kept in the fridge at 4°C in the meantime. The average of the cfu/ml (colony forming units per ml) from at least three different diluted suspensions was calculated for each diluted suspension using:

$$cfu/ml = (\frac{counted cfu}{0.01 ml}) * dilution factor$$

*Insect culture*

*Tenebrio molitor* larvae were originally provided by the company Ÿnsect (Evry, France). The insects were kept in a lab culture over more than six generations before the start of the experiments at the UK Centre for Ecology & Hydrology (Wallingford, United Kingdom). Adult *T. molitor* (40 females and 40 males) were kept in 750 ml containers (15 × 9.5 cm = 142.5 cm^2^) containing 50 g diet for four days. The diet consisted of wheat bran (96% w/w) and dried egg white (4% w/w). Additionally, the adults were fed with 5 g agar (1% w/v). Thereafter, the adults were removed and half of the containers containing eggs were transferred to the incubator set to high CO_2_, whereas the other half of the containers remained in the initial incubator at low CO_2_. The larvae received 10 g agar (1% w/v) twice a week starting one week after the removal of the adults and the number in each container was counted 17 days after the removal of the adults.

*Exposure of larvae to* B. thuringiensis

A suspension of *B. thuringiensis* was prepared as described above. In addition to estimating the spore concentration, the crystal concentration in the stock suspension was assessed by using a Bradford assay (see below). The larvae were divided into groups of 30 larvae and placed inside 60-ml cups (3.4 cm diameter) without lids 17 days after removal of the adults. The larvae were starved for 24 h in their respective CO_2_ treatments. Sixty ml cups containing the diet [wheat bran (96% w/w) and dried egg white (4% w/w)] and the spore suspensions were applied by adding 100 µl of 4 ×10^9^ spores/ml (4,999 and 1,678 µg/ml crystals in exp. 1 and 2, respectively) to 100 mg of diet in 60-ml cups. As a control 100 µl of dH_2_O was added to 100 mg of diet. The diet and the conidia suspension (or dH_2_O in the control) in each cup were mixed thoroughly with a sterile inoculation loop to make sure that the inoculum was homogeneously distributed. The 60-ml cups containing the diet and the spore suspensions (or dH_2_O in the control) were then incubated at either low or high CO_2_ for two days. After 2 days, the larvae were exposed; 100 µl of dH_2_O was added to each cup and the starved larvae were added to the respective cups. The larvae remained in the cups containing *B. thuringiensis* spores and crystals for two days in total.

*Exposure of larvae to* M. brunneum

*Metarhizium brunneum* (100 µl of 10^6^ conidia/ml) was grown on Petri dishes (9 cm diameter) containing 30 ml SDAY/4 media at either low or high CO_2_. After 14 days, conidial suspensions were prepared as described in the Supplementary methods, except that dH_2_O was added in the last washing step instead of TritonX-100 (0.05% v/v). Furthermore, conidial viability was assessed (see above). Larvae were starved as described in the section above (*Exposure of larvae to* B. thuringiensis). Thereafter, 100 µl of 10^8^ conidia/ml was added to 100 mg of diet in new 60-ml cups. As a control 100 µl of dH_2_O was added to 100 mg of diet. The diet and the conidia suspension (or dH_2_O in the control) in each cup were mixed thoroughly with a sterile inoculation loop to make sure that the inoculum was homogeneously distributed. The larvae were then put inside the respective cups, and they remained in the cups containing *M. brunneum* conidia for two days in total.

*Bradford assay*

A BAS (bovine albumin serum) solution (1 mg/ml) was prepared in dH_2_O. Eight 1-ml-cuvettes were prepared with different volumes of a NaOH solution (0.1 M; 800, 798, 796, 794, 792, 790, 785 and 780 µl). Thereafter, different volumes (0, 2, 4, 6, 8, 10, 15, and 20 µl, respectively) of the BAS solution as well as 200 µl of the Bradford reagent were added to the cuvettes resulting in a total volume of 1,000 µl in each cuvette. A piece of Parafilm^TM^ was put on each cuvette to avoid spillage and then all the cuvettes were vortexed and incubated at room temperature for 5 min. Afterwards, the OD (optical density) in each cuvette was measured at 595 nm using a WPA biowave CO8000 Cell Density Meter. From the obtained data points, the calibration curve was calculated using (y: OD, x: BAS volume in µl, a: slope, b: intercept):

$$y=ax+b$$

Either 2, 5, or 10 µl of the *B. thuringiensis* stock suspension, NaOH solutions (0.1 M; 798, 795 and 790 µl, respectively), as well as 200 µl of the Bradford reagent, were added to 1ml-cuvettes in triplicates. Thereafter, the OD in these cuvettes was measured at 595 nm and the average crystal concentration was calculated using:

$$toxin conc. in \mu g/mL = \frac{(OD-b)/a}{sample volume in mL}$$
